# The Parameter-Fitness Landscape of *lexA* Autoregulation in Escherichia coli

**DOI:** 10.1128/mSphere.00718-20

**Published:** 2020-08-19

**Authors:** Beverley C. Kozuch, Marla G. Shaffer, Matthew J. Culyba

**Affiliations:** a Department of Medicine, Division of Infectious Diseases, University of Pittsburgh School of Medicine, Pittsburgh, Pennsylvania, USA; Martin Luther University of Halle-Wittenberg Institute of Biology/Microbiology

**Keywords:** DNA damage, LexA, SOS response, autoregulation, feedback, transcription regulation

## Abstract

Feedback mechanisms are fundamental to the control of physiological responses. One important example in gene regulation, termed negative autoregulation (NAR), occurs when a transcription factor (TF) inhibits its own production through transcriptional repression. This enables more-rapid homeostatic control of gene expression. NAR circuits presumably evolve to limit the fitness costs of gratuitous gene expression. The key biochemical reactions of NAR can be parameterized using a mathematical model of promoter activity; however, this model of NAR has been studied mostly in the context of synthetic NAR circuits that are disconnected from the target genes of the TFs. Thus, it remains unclear how constrained NAR parameters are in a native circuit context, where the TF target genes can have fitness effects on the cell. To quantify these constraints, we created a panel of Escherichia coli strains with different *lexA-*NAR circuit parameters and analyzed the effect on SOS response function and bacterial fitness. Using a mathematical model for NAR, these experimental data were used to calculate NAR parameter values and derive a parameter-fitness landscape. Without feedback, survival of DNA damage was decreased due to high LexA concentrations and slower SOS “turn-on” kinetics. However, we show that, even in the absence of DNA damage, the *lexA* promoter is strong enough that, without feedback, high levels of *lexA* expression result in a fitness cost to the cell. Conversely, hyperfeedback can mimic *lexA* deletion, which is also costly. This work elucidates the *lexA*-NAR parameter values capable of balancing the cell’s requirement for rapid SOS response activation with limiting its toxicity.

**IMPORTANCE** Feedback mechanisms are critical to control physiological responses. In gene regulation, one important example, termed negative autoregulation (NAR), occurs when a transcription factor (TF) inhibits its own production. NAR is common across the tree of life, enabling rapid homeostatic control of gene expression. NAR behavior can be described in accordance with its core biochemical parameters, but how constrained these parameters are by evolution is unclear. Here, we describe a model genetic network controlled by an NAR circuit within the bacterium Escherichia coli and elucidate these constraints by experimentally changing a key parameter and measuring its effect on circuit response and fitness. This analysis yielded a parameter-fitness landscape representing the genetic network, providing a window into what gene-environment conditions favor evolution of this regulatory strategy.

## Abstract (precis)

Feedback mechanisms are critical to control physiological responses. In gene regulation, one important example, termed negative autoregulation (NAR), occurs when a transcription factor (TF) inhibits its own production. NAR is common across the tree of life, enabling rapid homeostatic control of gene expression. NAR behavior can be described in accordance with its core biochemical parameters, but how constrained these parameters are by evolution is unclear. Here, we describe a model genetic network controlled by an NAR circuit within the bacterium Escherichia coli and elucidate these constraints by experimentally changing a key parameter and measuring its effect on circuit response and fitness. This analysis yielded a parameter-fitness landscape representing the genetic network, providing a window into what gene-environment conditions favor evolution of this regulatory strategy.

## INTRODUCTION

**FIG 1:**
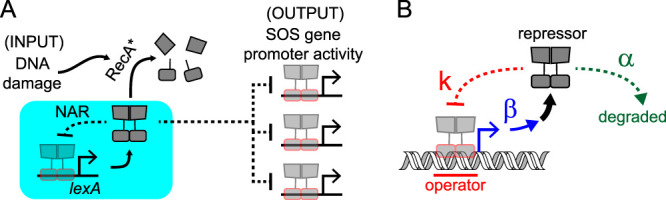
The SOS response and NAR circuit parameters. (A) SOS response schematic. LexA levels are maintained by the *lexA*-NAR circuit (cyan box), where the *lexA* gene itself is repressed by LexA. DNA damage leads to RecA activation (RecA*) and RecA*-induced LexA self-cleavage, which results in loss of LexA repressor activity and activation of LexA target gene promoters. (B) NAR circuit parameters. α is the first-order rate constant of repressor degradation (green), β is the rate of repressor synthesis (blue), and *k* is the repression constant (red), which describes the magnitude of repression for a given concentration of repressor and is related to repressor-operator binding affinity. NAR circuits with larger values of the parameter (β/α)/*k* have “stronger” autorepression (7).

To survive, cells must respond to fluctuations in their environment by rapidly altering their gene expression. Transcription regulation networks are a prevalent mechanism for temporal coordination of gene expression across the tree of life (1–6). In simple networks, a single transcription factor (TF) regulates multiple target promoters that control a set of functionally related genes. The environmental stimulus that modifies the activity of the TF can be viewed as the “input signal” for the circuit, and the resulting change in the promoter activity (PA) of the target genes of the TF can be viewed as the “output signal” (Fig. 1A). In Escherichia coli, most TFs are under autogenous control and exhibit negative autoregulation (NAR) (1, 6), where the TF also inhibits the promoter of its own gene. With NAR, perturbations below or above the steady-state concentration of the repressor are automatically compensated for by increased or decreased repressor synthesis rates, respectively. Thus, NAR is a mechanism for homeostatic control of repressor activity. Furthermore, with NAR, a stronger promoter can be utilized to achieve the same steady-state repressor concentration. This results in faster circuit “turn-off” kinetics (time to return to a repressed steady state) after the repressor has been inactivated by the input signal (7). Also, since repressor inactivation results in concurrent *de novo* repressor synthesis, NAR effectively increases the input dynamic range of a circuit (8). This aspect of NAR can scale network gene expression to a wider range of the input signal encountered by the cell. These features presumably make NAR advantageous for regulating gene networks where extensive, prolonged perturbations in repressor activity are detrimental, such as in the case of networks that encode toxic proteins. Despite the fundamental role of this regulatory motif, however, most studies have focused on synthetic NAR circuits that are detached from their downstream effector genes. Thus, we lack an understanding of how the biochemical parameters that dictate NAR circuit behavior are constrained by their fitness effects on the cell, the composition of TF target genes in the network, and the cellular environment.

The bacterial DNA damage repair pathway, or SOS response, is an ideal model system to study NAR, as the basic regulatory features are known (9, 10). In E. coli, the SOS response is comprised of a network of approximately 40 genes that encode DNA damage repair and tolerance activities, enabling the cell to survive genotoxic stress. The response is regulated by the LexA and RecA proteins. The LexA repressor serves as the master TF for all SOS genes by binding to specific operator sequences within their promoter regions and inhibiting their transcription, whereas RecA serves as the DNA damage sensor, stimulating LexA degradation during genotoxic stress. In the absence of DNA damage, LexA levels in the cell are high and SOS promoters are bound and repressed by LexA. In the setting of DNA damage, however, single-stranded DNA (ssDNA) is exposed at stalled replication forks. Exposure of ssDNA in the cell triggers RecA monomers to bind to and polymerize along the ssDNA, forming a nucleoprotein filament, referred to as RecA*, which contains a coprotease activity for LexA. RecA* induces LexA to undergo autoproteolysis, a self-cleavage reaction that inactivates its repressor activity, leading to derepression of SOS genes and activation of the SOS response. Most SOS gene promoters contain only a single-operator sequence, but some, such as *recN*, the plasmid-borne colicin genes, and *lexA*, contain multiple operator sequences (11). The presence of operator sites in the *lexA* promoter itself establishes an NAR circuit (12). This circuit impacts LexA levels in the cell and, therefore, the activation state of the entire SOS gene network (Fig. 1A). Unregulated expression of some SOS genes is toxic. For example, *sulA* encodes a protein that inhibits cell division and lack of LexA regulation is lethal to cells (13–15). Additionally, hyperregulation of SOS genes can also be detrimental, as bacteria that are incapable of activating the SOS response, by virtue of the presence of a noncleavable LexA protein, exhibit decreased survival after DNA damage (16–18). Therefore, we hypothesized that the full native SOS circuit context constrains *lexA-*NAR circuit parameters due to fitness effects on the cell. We sought to quantify this effect within the context of a previously described NAR-circuit model (7) and to examine its dependence on *sulA* activity and the degree of genotoxicity in the cellular environment.

Three main biochemical parameters describe an NAR circuit (7) (Fig. 1B), but only two of the parameters have been studied systematically in native circuit contexts. The first parameter, α, the first-order rate constant for the degradation of repressor (Fig. 1B, “α”), was examined in the SOS system using LexA variants with a range of autoproteolysis rates. Those studies showed that higher self-cleavage rates resulted in lower steady-state LexA levels and greater amounts of SOS activation (19, 20). The second parameter, β, or “promoter strength,” is the rate of mRNA synthesis in the unrepressed state of the promoter and dictates the maximum rate of synthesis of repressor protein (Fig. 1B, “β”). This parameter was investigated in the SOS system by using a tetracycline-inducible *lexA* promoter in a *lexA* deletion (*ΔlexA*) strain (P_tet_-*lexA*), enabling titratable control of *lexA* promoter activity without autorepression. As predicted by modeling (7), these experiments showed NAR that led to more-rapid “turn-off” kinetics, permitting faster exit from growth inhibition after DNA damage (21). In the present report, we investigate the third parameter, *k*, which is the defining feature of an NAR circuit, as it relates the magnitude of the promoter’s repression for a given repressor concentration (Fig. 1B, “k”). Of note, the mathematical framework derived to model NAR parameters (7) is based on a Michaelis-Menten model of promoter activity, *X*, where the unrepressed promoter activity, β, is modulated by the action of a repressor, *R*:

(1) $$X=\frac{\text{β}}{1+\frac{\left[R\right]}{k}}$$

In this formulation, [*R*] represents the concentration of the repressor and *k* represents the effective affinity of the repressor for the promoter (i.e., the concentration at half-maximal repression). At the steady-state repressor concentration, [*R*]_s_, the rate of repressor synthesis, *X*, is equal to its rate of degradation, α·[*R*]_s_:

(2) $$\frac{\text{β}}{1+\frac{{\left[R\right]}_{s}}{k}}=\text{α}{\left[R\right]}_{s}$$

Solving for *k* yields the following:

(3) $$k=\frac{{\left[R\right]}_{s}}{\frac{\text{β/α}}{{\left[R\right]}_{s}}-1}$$

To study the effect of specifically modulating *k* in the *lexA*-NAR circuit, we used site-directed mutagenesis to make a series of *lexA* promoters with mutations in the LexA operator sites. We engineered six mutant E. coli strains, exhibiting a wide range of LexA affinity at the *lexA* promoter, and characterized NAR circuit kinetics, input dynamic range, SOS functions, and fitness. The LexA steady-state concentrations of the strains ranged over 2 orders of magnitude, and our experiments showed that this impacted fitness and SOS functions at both extremes, thus revealing the constraints on NAR circuit parameters. We conclude by deriving a fitness landscape for the *lexA*-NAR circuit informed by our experimental data.

## RESULTS

### Construction of E. coli strains with a range of *k* values for the *lexA*-NAR circuit.

We sought to create a series of mutant *lexA* strains with different values of *k* and reasoned that this could be accomplished by introducing mutations into the promoter that are expected to specifically alter LexA-operator binding affinity. The *lexA* promoter has two known LexA operators located near the transcription start site (operators I and II) (22, 23) and a putative third, upstream operator (operator III) (24). Therefore, prior to deriving the full series of experimental constructs, we first determined the contribution of each operator to LexA-mediated repression. To do this, we introduced different combinations of a previously described T → C point mutation into the operator half-sites (see Fig. S1A in the supplemental material) (25), which severely abrogated LexA-operator affinity (Fig. S1B). To measure the effect of the mutations on LexA repressor activity, we utilized a green fluorescent protein (GFP)-reporter plasmid in which *gfp* is under the control of the *lexA* promoter (P*_lexA_*-*gfp*) (26) and analyzed expression in both the parental wild-type (wt; *lexA*^+^) and *ΔlexA* cells. Expression in *ΔlexA* cells serves as an important control, enabling measurement of the promoter’s inherent (LexA-independent) transcription activity. The mutations completely inactivated operators I and II but had no effect when introduced into operator III (Fig. S1C). In *ΔlexA* cells, promoter activity was relatively unaffected (Fig. S1D), confirming that the mutations caused a specific loss of LexA-mediated repression. Further analysis of operator III demonstrated that it was not functional for repression (Fig. S2); thus, we conclude that the *lexA* promoter has only two LexA operators.

**FIG 2:**
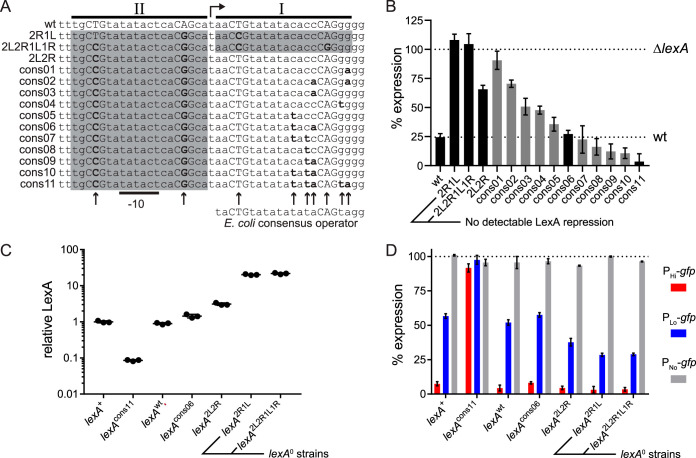
Construction of E. coli strains with a range of *k* values for the *lexA*-NAR circuit. (A) DNA sequences of mutant *lexA* promoter constructs. Each row of the alignment indicates a different *lexA* promoter construct. The high-LexA-affinity E. coli consensus operator sequence is given below operator I for comparison. Operator sequences (I and II) and the −10 site are indicated by horizontal lines, and the transcription start site is indicated with a bent arrow. Arrows indicate positions that were mutated in the alignment, and specific mutations are bolded. Conserved CTG motifs are indicated with capital letters, and operators shaded in gray were completely inactivated by mutation. (B) Promoter activity of mutant P*_lexA_*-*gfp* promoter constructs. Fluorescence intensity (GFP) measurements were acquired in *lexA*^+^ cells and normalized (% expression) to signal from an unrepressed control strain (*ΔlexA* = 100%). Plotted values and error bars represent means and standard deviations, respectively (*n* = 4). Black bars indicate promoters selected for strain construction. (C) Relative LexA levels in mutant *lexA* strains. LexA levels were quantified by immunoblotting and normalized to the signal obtained in the *lexA*^+^ control strain. Each circle represents a value from a replicate blot (*n* = 3). Horizontal lines and error bars represent geometric means and standard deviations, respectively. Error bars are not visible due to small variances between replicate values. (D) SOS target gene promoter activity in mutant *lexA* strains. Three different SOS reporter plasmid derivatives were used to assess promoter activity in the indicated strains: P_Hi_-*gfp* (red), P_Lo_-*gfp* (blue), and P_No_-*gfp* (gray) contain LexA operators with high, low, and no LexA affinity, respectively. Fluorescence intensity (GFP) measurements are normalized (% expression) to signal from an unrepressed control strain (*ΔlexA* = 100%). Plotted values and error bars represent means and standard deviations, respectively (*n* = 3). Strains that lack autorepression are also labeled “*lexA*^0^.”

10.1128/mSphere.00718-20.1 FIG S1 Mutational analysis of LexA operators on the *lexA* promoter. (A) Schematic of the *lexA* promoter and site-directed mutagenesis strategy. Each operator (I, II, or III) is composed of two half-sites containing a conserved CTG motif that is required for LexA binding. Site-directed mutagenesis was used to make a series of P*_lexA_*-*gfp* reporter plasmids containing a T → C base pair substitution in the CTG motif (CTG → CCG) in one or both half-sites of each operator. The locations of the RNA polymerase signal sequences (−10, −35) and transcription start site (arrow) are indicated. (B) Electrophoretic mobility shift assay (EMSA) of LexA binding to operators containing half-site mutations. Binding reaction mixtures contained 50 nM fluorophore-labeled dsDNA probe and no (lanes 2, 5, and 8), 100 nM (lanes 3, 6, and 9), or 500 nM (lanes 4, 7, and 10) recombinant LexA. Operator probes were based on the consensus DNA binding sequence of LexA and contained either no base pair substitutions (op^+^, lanes 2 to 4), a T → C base pair substitution in one half-site (op^-L^, lanes 5 to 7), or a T → C base pair substitution in both half-sites (op^-LR^, lanes 8 to 10). Lane 1, labeled ssDNA marker (M) for nonhybridized probe DNA. (C) Promoter activity of *lexA* operator mutants. Names of mutant promoters correspond to the locations of mutations in the schema of panel A. For example, the 1L promoter contains a single half-site mutation in the left (L) half-site of operator I and the 2L2R promoter contains both half-site mutations, left (L) and right (R), in operator II. Promoter activity values are given as a percentage of the value obtained in an unrepressed control (*ΔlexA* mutant). Plotted values and error bars represent means and standard deviations, respectively (*n* = 3). Alternating black and gray shading of bars is presented only a visual aid for specific operator groupings. (D) Raw promoter activity data (without normalization) for Fig. S1C. (E) Raw promoter activity data (without normalization) for Fig. 2B. The unnormalized values for the *ΔlexA* strain (panels D and E) allow comparison of the inherent (unrepressed) transcription activities of the promoters. Download FIG S1, PDF file, 0.6 MB. Copyright © 2020 Kozuch et al. 2020 Kozuch et al. This content is distributed under the terms of the Creative Commons Attribution 4.0 International license.

10.1128/mSphere.00718-20.2 FIG S2 The operator III site is not functional. (A) DNA sequence alignment of *lexA* promoters from closely related bacterial species showing that the CTG motifs of operator III are poorly conserved. The translation initiation codon (start), transcription start site (arrow), promoter signal sequences (−10, −35), and operator regions (I, II, and III) are indicated. Boxed residues indicate the location of the E. coli CTG motifs within each operator. (B) EMSA of LexA binding to the operator III DNA sequence. Binding reaction mixtures contained 50 nM fluorophore-labeled dsDNA probe and no (lanes 1 and 4), 500 nM (lanes 2 and 5), or 1,000 nM (lanes 3 and 6) recombinant LexA. Operator probes were based on the consensus DNA binding sequence of LexA (cons) or the E. coli operator III sequence (site III). (C) Installation of high-affinity LexA operators at site III had no effect on LexA-mediated repression. G → C or G → A substitution at position 15 of the operator III sequence or replacement of the operator III sequence with the consensus LexA binding sequence, all of which were expected to result in a higher level of expression of the LexA affinity operator, had no effect on LexA-mediated repression in either the wt or 2L2R1L1R promoter context. Download FIG S2, PDF file, 0.7 MB. Copyright © 2020 Kozuch et al. 2020 Kozuch et al. This content is distributed under the terms of the Creative Commons Attribution 4.0 International license.

The experiments described above yielded two constructs with a complete loss of LexA repression due to inactivation of both operators I and II (2R1L and 2L2R1L1R), as well as constructs with reduced repression due to inactivation of only a single operator (e.g., 2L2R) (Fig. 2A and B). Next, to create constructs with enhanced repression, we introduced mutations into operator I in a serial manner (using 2L2R as a starting template) that made it more similar to the E. coli consensus operator sequence (Fig. 2A), which is the DNA sequence that has the highest known affinity for LexA (27). We selected operator I for mutagenesis because, unlike operator II, it does not overlap highly conserved RNA polymerase (RNAP) binding sequences of the promoter, allowing us to make mutations that were unlikely to affect the promoter’s inherent transcription activity. To ensure this, we also measured the promoter activity of each construct in *ΔlexA* cells and found no major perturbations (Fig. S1E). As anticipated, promoter activity measurements in this “consensus” series (cons01 to cons11) decreased with greater similarity to the E. coli consensus operator sequence, yielding a collection of promoters with a range of levels of LexA repression that spanned that of the wt promoter (Fig. 2B).

We also further validated alteration of *k* by determining the promoter activation thresholds for a subset of promoters across this range of LexA repression (wt, 2L2R, cons06, and cons11). To do this, we measured the induction of promoter activity across a broad range of UV light-induced DNA damage and determined the UV dose that resulted in half-maximal activation (50% effective dose [ED_50_]) (Fig. S3A). Consistent with the expected alterations in *k*, the ED_50_ value for 2L2R was the lowest, that for cons11 was the highest, and that for cons06 was intermediate, with cons06 having the value closest to that of the wt promoter (Fig. S3B). Notably, the 2R1L and 2L2R1L1R promoters were excluded from this analysis because, consistent with a complete lack of repression, they did not show DNA damage induced by UV light. Finally, we note that a prior study of 14 mutant *recA* promoters, employing an analogous method, showed ED_50_ values that were highly correlated (*r* = 0.96, *P* < 0.0001) to biochemical measurements of LexA-operator binding (27). We conclude that the range of promoter activities that we report in Fig. 2B was due to differences in LexA binding affinity and that the range extends from the complete loss of LexA repression (2R1L and 2L2R1L1R), to an intermediate level of reduced repression (2L2R), to a level similar to wt repression (cons06), and finally to a level of repression exceeding that of the wt (cons11).

10.1128/mSphere.00718-20.3 FIG S3 Dose-response analysis of the wt, 2L2R, cons06, and cons11 promoters. (A) Dose-response analysis. *lexA*^+^ cells were transformed with each reporter plasmid, and the highest rate of GFP production (PA_peak_) was plotted for each indicated UV dose. Data points and error bars represent means and standard deviations, respectively (*n* = 2). The UV dose corresponding to 50% of maximal activity (50% effective dose [ED_50_]) was estimated by fitting the data to a dose-response model using nonlinear regression. Solid lines indicate the best fit of the regression, and vertical dotted lines indicate ED_50_ values for each promoter. (B) Plot of ED_50_ values. Data points and error bars represent the ED_50_ values and 95% confidence intervals as determined by nonlinear regression (see panel A). The horizontal line indicates the wt value. The value for each mutant was compared to the wt value using a two-tailed *t* test (*, *P* < 0.05; **, *P* < 0.01; ***, *P* < 0.001). Download FIG S3, PDF file, 0.3 MB. Copyright © 2020 Kozuch et al. 2020 Kozuch et al. This content is distributed under the terms of the Creative Commons Attribution 4.0 International license.

Having identified candidate promoters using the GFP-reporter plasmid system described above, we next introduced the wt, cons11, cons06, 2L2R, 2R1L, and 2L2R1L1R promoters into the endogenous *lexA* locus of the E. coli chromosome. Here, we refer to the parental wild-type and *lexA* deletion strains as the *lexA^+^* and *ΔlexA* strains, respectively, and refer to the resultant recombinant strains as the *lexA*^wt^, *lexA*^cons11^, *lexA*^cons06^, *lexA*^2L2R^, *lexA*^2R1L^, and *lexA*^2L2R1L1R^ strains. These strains harbor intact *lexA*-NAR circuits that are fully integrated into the native SOS gene network. The *lexA*^2R1L^ and *lexA*^2L2R1L1R^ strains are also fully integrated, but their *lexA*-NAR circuits lack autoregulation because LexA does not repress their mutant *lexA* promoters. Below, we refer to the latter two strains collectively as *lexA*^0^ strains to denote the complete absence of autorepression. We selected the set of promoters for strain construction described above since they represent a range for the parameter *k* in this system with values between the two LexA repression extremes: *lexA*^0^ (no repression, *k* → ∞) and *ΔlexA* (no expression, *k* → 0). The ranking of the strains in terms of the expected values for *k* is as follows: *ΔlexA* < *lexA*^cons11^ < *lexA*^wt^ ∼ *lexA*^cons06^ < *lexA*^2L2R^ < *lexA*^0^. Of note, the double-operator configuration of the wt promoter is speculated to facilitate positive cooperative binding interactions between LexA dimers (11, 20, 22, 23, 28). Therefore, we selected the cons06 construct for strain construction for the additional reason that its degree of LexA repression was similar to that seen with the wt promoter and yet it contains only a single operator, leading to our reasoning that a direct comparison of *lexA*^cons06^ to *lexA*^wt^ would provide insight into the importance of the double-operator configuration (see Discussion).

Changing the value of *k* independently of other NAR circuit parameters is predicted to change the steady-state concentration of the repressor (see equation 3), which, in turn, would change the expression of repressor target genes. Therefore, to characterize and validate the mutant strains, we first measured their relative LexA levels by quantifying LexA immunoblots (Fig. 2C). As expected, no LexA immunoblot signal was detected in *ΔlexA* cells (Fig. S4A) and *lexA*^wt^ cells exhibited LexA levels similar to those seen with the parental *lexA*^+^ strain. LexA levels in *lexA*^cons11^ were close to the limit of detection (Fig. S4B), at about one-tenth that of *lexA*^wt^. This represents a significant reduction, as it is comparable to the amount of LexA depletion that resulted from 30 J/m^2^ of UV-induced DNA damage (Fig. S4C). LexA levels in *lexA*^cons06^ were similar to those seen with *lexA*^wt^, and levels in *lexA*^2L2R^ were between those seen with the *lexA*^wt^ and the *lexA*^0^ strains. Consistent with the absence of autorepression, the *lexA*^0^ strains had the highest LexA levels, which were about 23-fold higher than shown by the *lexA*^wt^ cells. Next, to determine the impact on target gene expression, we transformed the mutant strains with the following three different SOS GFP-reporter plasmids, which were based on the *recA* promoter (27): one plasmid had a promoter with a high affinity for LexA (P_Hi_-*gfp*), another plasmid had a promoter with low affinity for LexA (P_Lo_-*gfp*), and, as a control, we also included a promoter with no affinity for LexA (P_No_-*gfp*). We then compared the levels of promoter activity between the strains (Fig. 2D). As expected, we observed near-maximal activity (93% to 101%) with the P_No_-*gfp* reporter plasmid in all strains, signifying a complete lack of repression of *gfp* expression. For the P_Lo_-*gfp* reporter plasmid, we observed similar levels of activity for *lexA*^+^, *lexA*^wt^, and *lexA*^cons06^ (52% to 58%), decreased activity for *lexA*^2L2R^ (38%), further reduced activity for the *lexA*^0^ strains (29%), and near-maximal activity for *lexA*^cons11^ (98%). For the P_Hi_-*gfp* reporter plasmid, we observed <9% activity for all of the strains except *lexA*^cons11^, which displayed 92% activity. Overall, we found that target gene expression was inversely correlated with the amount of LexA present in the cell and conclude that the altered steady-state LexA concentrations of the mutant strains affected the activity of SOS gene promoters with both high and low LexA affinity. Interestingly, promoter activity was near-maximal in the *lexA*^cons11^ strain even after transformation with the P_Hi_-*gfp* plasmid, a very-low-copy-number plasmid containing the highest-affinity LexA operator sequence. This raised the possibility that *lexA*^cons11^ may have a constitutively active SOS response (see below).

10.1128/mSphere.00718-20.4 FIG S4 LexA immunoblots. (A) LexA immunoblot signals of mutant strains. Prior to gel loading, samples from the *lexA*^2L2R^, *lexA*^2R1L^, and *lexA*^2L2R1L1R^ strains were diluted by factors of 2, 4, and 4, respectively. The relative total protein loading level in each lane was determined by quantifying results obtained with a Coomassie-stained SDS-PAGE gel (numbers displayed below the gel image). (B) Immunoblot signal of *ΔlexA* (lanes 2 to 5) and *lexA*^cons11^ (lanes 6 to 9) strains at long exposure time. Lanes 1 and 10, molecular weight markers (M). (C) LexA depletion of *lexA*^+^ strain, as determined by immunoblotting, after UV doses of 1, 3, and 30 J/m^2^. Download FIG S4, PDF file, 1.9 MB. Copyright © 2020 Kozuch et al. 2020 Kozuch et al. This content is distributed under the terms of the Creative Commons Attribution 4.0 International license.

Having validated significant alterations to the parameter *k* in the mutant strains, we next took advantage of our unique experimental system to quantify *k* for each strain. In the strains that we derived, the quantity β/α of the *lexA*-NAR circuit is constant and can be inferred from the LexA steady-state concentration of the *lexA*^0^ strains, [LexA]_0_, as follows:

(4) $$[\text{LexA}{]}_{0}=\frac{\beta }{\alpha }$$

Equation 4 enables [LexA]_0_ to be treated as a constant and substituted for β/α within equation 3. The ability to determine [LexA]_0_ in this manner allows *k* to be solved in terms of the LexA steady-state concentration of any strain where autorepression remains intact, [LexA]_s_, as follows:

(5) $$k=\frac{{\left[\text{LexA}\right]}_{s}}{\frac{{\left[\text{LexA}\right]}_{0}}{{\left[\text{LexA}\right]}_{s}}-1}$$

Thus, to quantify *k* for each strain, we used our measurements of LexA levels (Fig. 2C) as the input for equation 5. For the value of [LexA]_0_, we averaged the two *lexA*^0^ strain measurements since both model the complete absence of autorepression (as shown below, we also grouped measurements from the *lexA*^0^ strains together for this reason). We found that the value for *k* spanned 3 orders of magnitude among the strains with intact autorepression (Table 1).

**TABLE 1:** Calculated values for the repression constant, *k*^*a*^

Strain	[LexA]_s_ (95% CI)	[LexA]_0_ (95% CI)	*k* (95% CI)
*lexA* ^+^	1.1 (0.9–1.3)		0.06 (0.03–0.08)
Δ*lexA*	0 (NA)		0 (NA)
*lexA* ^cons11^	0.10 (0.08–0.11)		0.0004 (0.0003–0.0005)
*lexA* ^wt^	1.0 (0.8–1.2)		0.04 (0.03–0.06)
*lexA* ^cons06^	1.6 (1.1–2.1)		0.12 (0.04–0.20)
*lexA* ^2L2R^	3.4 (2.7–4.2)		0.60 (0.33–0.87)

*lexA*^0^ strains			
*lexA*^2R1L^		22 (20–25)	∞ (NA)
*lexA*^2L2R1L1R^		24 (21–28)	∞ (NA)

a Equation 5 was used to calculate *k* values with [LexA]_0_ = 23, which represents the average of the *lexA*^2R1L^ and *lexA*^2L2R1L1R^ [LexA]_s_ values. [LexA]_s_ = 0 for the *ΔlexA* strain due to genetic deletion and *k* → 0 in this context. Similarly, for the *lexA*^2R1L^ and *lexA*^2L2R1L1R^ strains, where no autoregulation exists, *k* → ∞. CI, confidence interval; NA, not applicable.

### Circuit input sensitivity, dynamic range, and target gene expression kinetics of mutant strains.

To determine the effect of *k* on input sensitivity, dynamic range, and target gene expression kinetics, we measured the promoter activity of the P*_lexA_*-*gfp* and P_Hi_-*gfp* reporter plasmids (i.e., the circuit “output”) in the mutant strains as a function of time over a range of different DNA damage doses (i.e., the circuit “input”). We chose the P*_lexA_*-*gfp* and P_Hi_-*gfp* promoters for this analysis because they have low LexA affinity and high LexA affinity, respectively, and have expression kinetics that are representative of “early” and “late” SOS genes, respectively (27). We excluded the *ΔlexA* and *lexA*^cons11^ strains from this analysis because they display constitutive SOS GFP-reporter activity and the circuit could not be further induced. The remaining strains were inducible and have values for *k* and [LexA]_s_ that are equal to or greater than wild type. We hypothesized that the higher values for [LexA]_s_ in the mutant strains would be an impediment to SOS activation and, therefore, decrease circuit sensitivity, increase dynamic range, and slow target gene expression kinetics.

**FIG 3:**
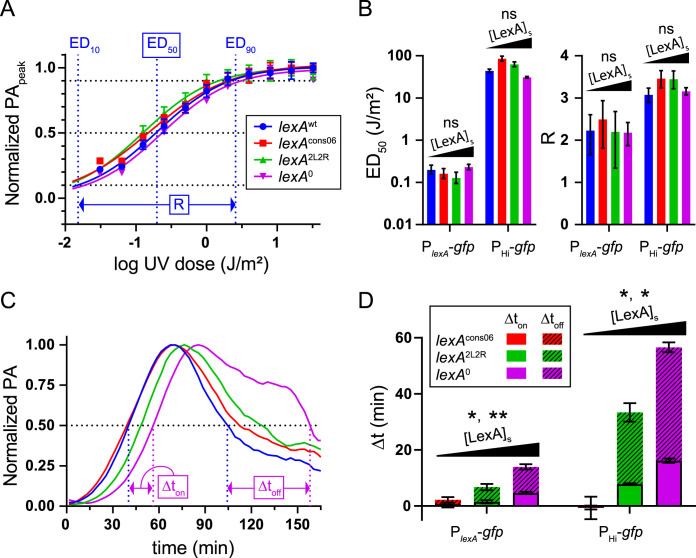
Circuit input sensitivity, dynamic range, and expression kinetics of mutant *lexA* strains. (A) Dose-response analysis of circuit output. Normalized peak promoter activity values (PA_peak_) are plotted as a function of UV dose for each strain. Values for P*_lexA_*-*gfp* are shown. Data points and error bars represent means and standard deviations, respectively (*n* = 2 to 4). Lines indicate the best fit as determined by nonlinear regression. Horizontal black dotted lines indicate 10% and 90% of maximum PA. Vertical blue dotted lines indicate the UV dose which resulted in 10% (ED_10_), 50% (ED_50_), and 90% (ED_90_) of PA_peak_ for the *lexA*^wt^ strain. The horizontal blue double-arrow line indicates the input dynamic range (*R*) for the *lexA*^wt^ strain, where *R* = log(ED_90_/ED_10_) = log(81)/H and H represents the value of the Hill slope for the best-fitted line. (B) Plots of ED_50_ and R for each strain, as determined by nonlinear regression. The plotted values and error bars represent the means and 95% confidence intervals from two different LexA-regulated promoters, P*_lexA_*-*gfp* and P_Hi_-*gfp*. Black triangles indicate that the strains indicated at the bottom are ordered from left to right by increasing value of [LexA]_s_ and that linear regression was performed (ns, *R*^2^ < 0.5 and *P* > 0.05, see Fig. S5A and B). (C) Representative “promoter activity” versus “time” plot of P_Hi_-*gfp* output after administration of a UV dose of 32 J/m^2^. The horizontal dotted line indicates 50% PA_peak_. Vertical dotted lines indicate *t*_on_ and *t*_off_ for *lexA*^wt^ (blue) and *lexA*^0^ (magenta). The double-ended arrows point to the *t*_on_ and *t*_off_ values used to calculate Δ*t*_on_ and Δ*t*_off_ for *lexA*^0^. (D) Target gene expression kinetics. Means and standard errors for Δ*t*_on_ (solid) and Δ*t*_off_ (striped) from 11 different UV doses are shown from two different LexA-regulated promoters (P*_lexA_*-*gfp* and P_Hi_-*gfp*) for each mutant strain. Black triangles indicate that the strains indicated at the bottom are ordered from left to right by increasing value of [LexA]_s_ and that linear regression was performed (*, *P* < 0.05; **, *P* < 0.01 [for Δ*t*_on_ and Δ*t*_off_]; see Fig. S5C).

First, to understand the effect of *k* on the input sensitivity and dynamic range of the circuit, we plotted the highest promoter activity value obtained (PA_peak_) as a function of UV dose and analyzed the dose-response curves. We used the value for the UV dose that half-maximally activated the promoter (ED_50_) as a measure of circuit “input sensitivity” and the difference between the ED_90_ and ED_10_ values (*R*) as a measure of “input dynamic range” (Fig. 3A). Consistent with past studies (27), we found that the ED_50_ values for P_Hi_-*gfp* induction were higher than those of for P*_lexA_*-*gfp* induction (Fig. 3B, left), indicating that a larger UV dose is required to activate the higher-LexA-affinity promoter. However, here, our data newly enabled us to test the hypothesis that elevated [LexA]_s_ values decrease circuit sensitivity (i.e., increase ED_50_ values), but we found no significant association between ED_50_ and [LexA]_s_ values (Fig. S5A). Similarly, we found that the *R* value for the P_Hi_-*gfp* promoter was higher than that for the P*_lexA_*-*gfp* promoter but, again, that there was no significant association between *R* and [LexA]_s_ (Fig. S5B). We conclude that increasing [LexA]_s_ by altering the value of *k*, or even eliminating autorepression entirely, does not impact the input sensitivity or dynamic range in this system.

10.1128/mSphere.00718-20.5 FIG S5 Regression analysis of data presented in Fig. 3B and D. (A) Plot of LogED_50_ versus Log[LexA]_s_. (B) Plot of R versus Log[LexA]_s_. (C) Plot of Δt versus Log[LexA]_s_. Data points represent mean values. The lines of best fit from linear regression are shown, and R^2^ and *P* values are displayed next to each line. Download FIG S5, PDF file, 0.3 MB. Copyright © 2020 Kozuch et al. 2020 Kozuch et al. This content is distributed under the terms of the Creative Commons Attribution 4.0 International license.

Next, to understand the effect of *k* on target gene expression kinetics, we measured the amount of time that was required for the promoter to reach 50% of its maximal signal activity (*t*_on_) and then to return to 50% of its maximal activity (*t*_off_) for each DNA damage dose. To understand the degree to which the mutant strain value deviated from that determined for the wild-type strain, we calculated Δ*t*_on_ and Δ*t*_off_, representing the difference between the value determined for the mutant strain and that representing the wild-type strain (Fig. 3C). We found that the Δ*t*_on_ and Δ*t*_off_ values were relatively invariant with respect to DNA damage dose, and so we plotted the average values for comparison (Fig. 3D). In contrast to the ED_50_ and *R* values, which exhibited no correlation with [LexA]_s_, we found that the Δ*t*_on_ and Δ*t*_off_ values were significantly correlated with [LexA]_s_ (Fig. S5C). The highest Δ*t*_on_ values were exhibited by *lexA*^0^ cells (Δ*t*_on_ = 5 min for P*_lexA_*-*gfp* and 16 min for P_Hi_-*gfp*); thus, our analysis quantified this extreme state of the *lexA-*NAR circuit. Consistent with an overall slower DNA repair process, the Δ*t*_off_ values were similar to or greater than the Δ*t*_on_ values for each strain. Taking the results together, analysis of the *lexA*^cons11^ mutant revealed that decreasing the value of *k* (to below the wild-type value) in this system collapsed the input dynamic range due to a [LexA]_s_ value that is ∼10% that of the wild-type strain. Conversely, increasing the [LexA]_s_ value by altering the value of *k* reduced the duration of “turn-on” kinetics of target gene expression by as much as 16 min. We infer that it takes the cell more time to inactivate the larger amount of LexA present. Interestingly, higher LexA steady-state levels had no effect on the circuit’s input sensitivity or dynamic range. We conclude that additional SOS regulatory features are able to compensate for higher LexA levels and, eventually, to meter out the same circuit output at any level of DNA damage. Thus, the primary effect of increasing the value of *k* is to slow the turn-on kinetics of the SOS response.

### DNA damage survival and fitness of mutant strains.

**FIG 4:**
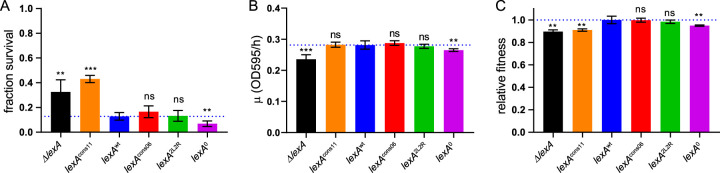
DNA damage survival and fitness of mutant *lexA* strains. (A) Survival after a UV dose of 40 J/m^2^. The dotted line indicates the value for *lexA*^wt^. (B) Growth rates. Plotted values and error bars represent the maximum growth rates (μ) and 95% confidence intervals determined by analysis of growth curves (Fig. S7) using nonlinear regression. (C) Relative fitness in competitive growth. Fitness values are normalized to *lexA*^wt^ (dotted line = 1). For panels A and C, plotted values and error bars represent means and standard deviations (*n* = 3 to 6). The mean for each mutant strain was compared to the mean for *lexA*^wt^ using a two-tailed *t* test (ns, *P* > 0.05; **, *P* < 0.01; ***, *P* < 0.001).

Activation of the SOS response results in enhanced survival of DNA damage due to temporally ordered induction of expression of “early” DNA repair genes involved in nucleotide excision repair (NER) and “late” damage-tolerant DNA polymerases capable of error prone translesion synthesis (TLS) (9). Therefore, to test for alterations in SOS effector function, we first measured survival rates under conditions of UV light exposure and the ability of the mutants to induce mutagenesis, which is the signature of TLS activity. We found that the rate of survival that was exhibited by the *ΔlexA* and *lexA*^cons11^ strains after a UV dose of 40 J/m^2^ was approximately 3-fold-higher than that exhibited by the *lexA*^wt^ strain (Fig. 4A). This finding suggests that preinduction of the SOS DNA damage repair and tolerance activities in the *ΔlexA* strain provides additional UV tolerance and that the *lexA*^cons11^ strain is similar to the *ΔlexA* strain in this regard. The ability of the *lexA*^cons06^ and *lexA*^2L2R^ strains to survive this insult was similar to that shown by the *lexA*^wt^ strain. In contrast, *lexA*^0^ cells showed approximately 2-fold-lower survival than the *lexA*^wt^ cells, revealing a defect in SOS effector function. The defect was not due to an inability to induce damage-tolerant polymerases, however, as *lexA*^0^ cells had the same level of induced mutagenesis as *lexA*^wt^ (Fig. S6). This result is consistent with our finding of *lexA*^0^ cells having the same input sensitivity and dynamic range as *lexA*^wt^ cells and suggests that the UV survival defect is primarily due to slower SOS turn-on kinetics.

10.1128/mSphere.00718-20.6 FIG S6 UV-induced mutagenesis of mutant strains. Data are from the UV survival experiment represented in Fig. 4A (UV dose = 40 J/m^2^). Here, mutagenesis was scored by counting rifampin-resistant (Rif^r^) colonies and calculating the Rif^r^ frequencies in each culture. The increase in Rif^r^ frequency after irradiation is plotted as the logarithm of the Rif^r^ frequency ratio of irradiated and nonirradiated cultures. Plotted values and error bars represent means and standard deviations (*n* = 3 to 6). The mean of the results determined for each mutant strain was compared to the mean of the results determined for *lexA*^wt^ using a two-tailed *t* test (ns, *P* > 0.05; **, *P* < 0.01; ***, *P* < 0.001). Download FIG S6, PDF file, 0.3 MB. Copyright © 2020 Kozuch et al. 2020 Kozuch et al. This content is distributed under the terms of the Creative Commons Attribution 4.0 International license.

10.1128/mSphere.00718-20.7 FIG S7 Growth curves of mutant strains. Growth in LB medium was monitored by OD_595_ through time. Data points represent mean values (*n* = 3), and lines of best fit are derived from curve fitting by nonlinear regression. Download FIG S7, PDF file, 0.3 MB. Copyright © 2020 Kozuch et al. 2020 Kozuch et al. This content is distributed under the terms of the Creative Commons Attribution 4.0 International license.

We next measured growth kinetics and relative fitness levels in the absence of exogenous DNA damage. Monitoring growth of each strain in monoculture, we found the *ΔlexA* and *lexA*^0^ strains displayed lower growth rates than the other strains (Fig. 4B), with the *ΔlexA* strain exhibiting the slowest growth and also lower density in stationary phase (Fig. S7). To quantify relative fitness levels, each mutant strain was also competed against the *lexA*^+^ parental strain in coculture using a competitive growth assay. Similarly to their growth in monoculture, the *ΔlexA* and *lexA*^0^ strains showed decreased fitness; however, this assay also revealed a fitness defect in the *lexA*^cons11^ strain (Fig. 4C) that was not made apparent by monitoring growth in monoculture (Fig. 4B). We also obtained a similar result when a subinhibitory amount of the DNA strand cross-linking agent mitomycin C (MMC) was added to the media (Fig. S8). These results contrast with those from the UV survival experiments described above, where the *ΔlexA* and *lexA*^cons11^ strains displayed a survival advantage over the *lexA*^wt^ strain, demonstrating that this advantage is restricted to high amounts of DNA damage. We conclude that lowering the [LexA]_s_ level by altering *k* can increase fitness after a lethal dose of DNA damage but results in decreased fitness with lower, subinhibitory amounts of DNA damage. Conversely, raising the [LexA]_s_ level by altering *k* decreases fitness whether in the presence or the absence of DNA damage.

10.1128/mSphere.00718-20.8 FIG S8 Relative fitness in the presence of 1 μg/ml mitomycin C. Fitness values are normalized to *lexA*^wt^ values. Plotted values and error bars represent means and standard deviations (*n* = 3 to 6). The mean of the results determined for each mutant strain was compared to the mean of the results determined for the *lexA*^wt^ strain using a two-tailed *t* test (ns, *P* > 0.05; *, *P* < 0.05; **, *P* < 0.01). Download FIG S8, PDF file, 0.3 MB. Copyright © 2020 Kozuch et al. 2020 Kozuch et al. This content is distributed under the terms of the Creative Commons Attribution 4.0 International license.

Our analysis of the *lexA*^cons11^ strain showed that lowering the value of *k* can reduce the level of [LexA]_s_ enough in terms of target gene expression, UV survival, and fitness in competitive growth assays to mimic the *ΔlexA* strain. However, we noted that *lexA*^cons11^ differed from the *ΔlexA* strain in other assays: it displayed normal growth in monoculture (Fig. 4B) and yielded intermediate phenotypes for UV-induced mutagenesis (Fig. S6) and relative fitness under subinhibitory MMC conditions (Fig. S8). These results show that some degree of SOS gene regulation by LexA remained in *lexA*^cons11^ cells. The mutant *lexA* strains studied here were constructed in a *ΔlexA* background since *ΔlexA* strains are not viable in a *sulA*^+^ background. This conditional lethality is due to the inhibition of cell division by the SulA protein in *ΔlexA* strains, as *sulA* is repressed by LexA. However, it is unknown whether the complete absence of LexA protein (*ΔlexA*) is necessary for lethality or, instead, if very low [LexA]_s_ levels (∼10% of wild-type levels), as observed in the *lexA*^cons11^ strain, are sufficient for *sulA* repression and viability. Therefore, we attempted to complement the mutant strains with *sulA*^+^ on a very-low-copy-number plasmid by transformation. As expected, we readily obtained *sulA*^+^ transformants in the *lexA^+^* strain but obtained no transformants in the *ΔlexA* strain. Transformation of *sulA*^+^ into the mutant strains occurred with a level of efficiency similar to that seen with the *lexA*^+^ strain, with the notable exception being the *lexA*^cons11^ strain, where we also did not obtain any transformants (see Table S1 in the supplemental material). This result demonstrates how the presence of a toxic target gene in a network can further constrain NAR circuit parameters. The low value of *k* leads to persistently low LexA levels and does not permit growth, phenocopying *lexA* deletion. We conclude that the presence of *sulA*^+^ in the SOS gene network of E. coli imposes a potent constraint on the range of permissible *lexA-*NAR circuit parameter values.

10.1128/mSphere.00718-20.9 TABLE S1 Transformation of mutant strains with *sulA*^+^. Values and ranges indicate the means and standard errors, respectively, of raw colony counts obtained from independent transformations (*n* = 3) with the pBCK005 plasmid (vector control) or the pBCK059 plasmid (*sulA*^+^). Download Table S1, PDF file, 0.2 MB. Copyright © 2020 Kozuch et al. 2020 Kozuch et al. This content is distributed under the terms of the Creative Commons Attribution 4.0 International license.

### Fitness landscape of *lexA*-NAR circuit parameters.

**FIG 5:**
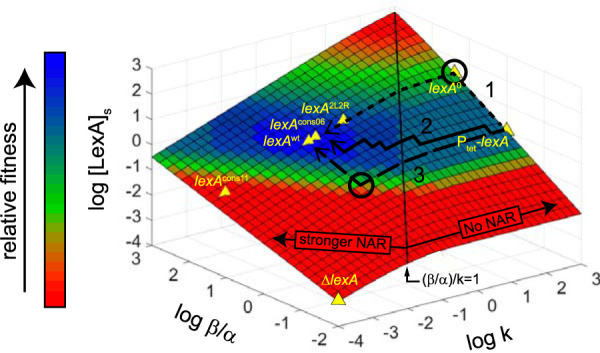
Parameter-fitness landscape of the *lexA*-NAR circuit. The 3D plot represents log_10_ transformation of data from equation 6, with surface colorization denoting relative fitness (see legend). The plot models a low-genotoxicity environment in a *sulA*^+^ background. The vertical black line represents (β/α)/*k* = 1. For (β/α)/*k* values of <1 (No NAR), the circuit is nonautogenous and [LexA]_s_ values are independent of *k*. For (β/α)/*k* values of >1 (stronger NAR), autorepression strength increases with larger values and significantly impacts circuit response times (7). Locations of *lexA*-NAR mutant strains on the plot are indicated with yellow triangles. Strains with extreme parameter values were placed on the edges of the surface, a location with fitness equivalent to the actual parameter values. For example, *k* → 0 for the *ΔlexA* strain since [LexA]_s_ = 0 and *k* → ∞ for the *lexA*^0^ and P_tet_-*lexA* strains since their promoters lack LexA binding sites. The fitness difference between *lexA*^wt^ and P_tet_-*lexA* was demonstrated previously (21). Hypothetical evolutionary paths from P_tet_-*lexA* → *lexA*^wt^ are shown with lines with thick black short dashes (pathway 1), solid lines (pathway 2), and lines with long dashes (pathway 3). Circles highlight fitness cost excursions. Pathway 1 models serial promoter mutations, followed by serial operator mutations, resulting in a fitness cost excursion. Pathway 2 models alternating promoter and operator mutations along a path of continuous fitness increases. Pathway 3 models serial operator mutations, followed by serial promoter mutations, resulting in a fitness cost excursion.

The analysis described above enabled us to derive an NAR parameter-fitness landscape using our experimental data in combination with the previously developed mathematical framework (7). To do this, we utilized an equation that relates the steady-state concentration of the repressor, [LexA]_s_, to the parameters *k* and β/α to construct a three-dimensional (3D) surface describing the relationships among these three parameters (see Materials and Methods, equation 6). Then, we used our experimental data to plot our mutant strains onto this surface and also to overlay a fourth “relative fitness” parameter onto the surface using a color gradient (Fig. 5). The mutant E. coli strains constructed in the present study were plotted on the same surface isoline of the landscape [defined by log_10_(β/α) = 1.3], since they share a value for this parameter. *lexA*^cons11^ occupies the red (nonviable) portion of the plot because the low value of [LexA]_s_ in this strain results in lethal expression of *sulA*. In the plot, the strains span the entire fitness landscape, from *lexA*^cons11^ (red = nonviable) to *lexA*^wt^ and *lexA*^cons06^ (blue = high fitness) and, finally, to *lexA*^0^ (green = intermediate fitness). Additionally, this framework accommodates the results from a prior study of an E. coli mutant (Fig. 5, “P_tet_-*lexA*”) that completely lacked autoregulation but that had the same LexA steady-state levels as a wild-type strain due to a lower value of β for the promoter (21). This mutant strain displayed a growth defect after sublethal DNA damage and is therefore shown with a fitness level lower than that of the wild-type strain in the plot. In this framework, alterations of *lexA*-NAR circuit parameters are the result of specific changes in DNA that alter the key biochemical reactions of NAR. For example, mutations in operator DNA or the portion of *lexA* encoding its DNA binding domain can alter the value of *k* (27, 29, 30), mutations in the portion of *lexA* encoding its protease domain can alter the value of α (19, 31–33), and mutations in the *lexA* promoter that affect RNAP activity can alter the value of β. In contrast, the coloration of the plot is modified by the environment, genetic background, and epistatic interactions with the SOS gene network and, therefore, the landscape depicted here represents only a snapshot of the current experimental conditions. In summary, the plot captures the fitness effects of altering the main biochemical parameters of the *lexA*-NAR circuit, yielding a basic framework suitable to consider the fitness constraints of different evolutionary paths on the landscape (see Discussion).

## DISCUSSION

Prior studies of NAR circuit behavior have largely focused on isolated synthetic circuits to validate mathematical models of NAR, demonstrating how NAR circuit parameters affect circuit shutoff kinetics and input dynamic range. However, relatively little parameter space has been analyzed in the context of a native circuit, where the TF still controls the expression of its target genes. In one native circuit study that also used the SOS response as a model, a *ΔlexA* strain was complemented with the *lexA* gene under the regulation of the tet promoter (P_tet_-*lexA*), providing a circuit that completely lacked autoregulation but that still exhibited LexA steady-state concentrations similar to those seen with the wild-type strain (21). This study allowed comparison of autogenous and nonautogenous control under conditions where, in the absence of a DNA damage input, the two genetic networks were similarly repressed. After DNA damage, however, the two strains displayed the same circuit turn-on time, but the P_tet_-*lexA* strain had a longer shutoff time which was associated with a growth defect (see Fig. 5, “*lexA*^wt^” versus “P_tet_-*lexA*”). This study supports the hypothesis that NAR represents an adaptation for increasing fitness by speeding up circuit shutoff kinetics. However, a more systematic analysis of the full parameter space was lacking; therefore, it remained unclear how, and to what extent, NAR circuit parameters are more globally constrained by their fitness effects on the cell. To address this issue, we created a novel series of E. coli mutants that spanned the full range of the *lexA*-NAR circuit parameter, *k*, and measured circuit output, SOS functions, and cellular fitness. These data allowed us to extend the mathematical framework for NAR to include a complete parameter-fitness landscape, offering new insights into how *lexA*-NAR parameters are constrained by their fitness effects on the cell.

First, we found that *lexA*-NAR circuits with higher values of *k*, resulting in higher levels of [LexA]_s_, did not have altered input sensitivity or dynamic range but did have slower turn-on kinetics. In *lexA*^0^ strains, where the [LexA]_s_ value is 23-fold higher than in the wild-type strain, this resulted in reduced survival of DNA damage, which we attribute to the turn-on kinetics of their LexA target genes being 5 to 16 min slower than the kinetics of the wild-type strain. Of note, this timescale is consistent with a prior report from a study that analyzed the ability of delayed SOS activation to rescue cells from lethal DNA damage (34). Thus, although it takes the SOS system more time to degrade the higher amount of LexA present in these mutant strains, the DNA damage sensing reaction performed by RecA (i.e., ssDNA + RecA ⇌ RecA*) apparently compensates appropriately, eventually inducing enough degradation of LexA to meter out the same SOS activities as in the wild-type case. However, the slower repair process is associated with a fitness cost, showing that slow turn-on kinetics is a mechanism that constrains the parameters of the *lexA*-NAR circuit. We also observed a fitness cost with *lexA*^0^ cells in the absence of exogenous DNA damage. Under these conditions, persistently high levels of LexA may hyperrepress the SOS genes that are needed under nonstressful conditions. Alternatively, high LexA levels may interfere with cellular metabolism, either by nonspecific DNA binding or simply due to the metabolic burden of its higher rates of synthesis and degradation. Regardless of the precise mechanism, the fitness defect displayed by *lexA*^0^ cells under these conditions reveals that the *lexA-*NAR circuit has a value for β/α that is toxic in the absence of negative feedback. If one considers the evolution of a repressor gene’s promoter toward faster circuit shutoff kinetics, speed could be attained by simply increasing the value of β/α (Fig. 5, pathway “1”). This could be achieved, for example, by promoter mutations that increase RNAP activity. However, our data show that, for the SOS system, this strategy can reduce fitness (Fig. 5, *lexA*^0^). Thus, for the circuit to evolve shorter shutoff times without incurring a fitness cost, the value of *k* must also be altered in proportion to the change in β/α (Fig. 5, pathway “2”).

Second, we found that the value of *k* is also constrained by fitness effects at lower values. The *lexA*^cons11^ strain, where the value of [LexA]_s_ is 10-fold lower than the value measured for the wild-type strain, mimicked the constitutive SOS phenotypes of the *ΔlexA* strain, including conditional lethality with *sulA*^+^. Thus, lowering the value of *k* can result in a loss of input dynamic range and a fitness cost due to derepression of toxic genes. Again, considering the evolution of a repressor gene’s promoter toward faster circuit shutoff kinetics, speed can be attained by simply decreasing the value of *k* (Fig. 5, pathway “3”). This could be achieved, for example, by mutation of the *lexA* operators. However, this pathway is also constrained by fitness effects, which are more severe in the presence of *sulA*^+^. Thus, in this case, for the circuit to evolve its shutoff time independently of toxic gene expression, the value of β/α must also be altered in proportion to the change in *k* (Fig. 5, pathway “2”).

Importantly, fitness landscapes in nature are not static and can be altered by numerous different mechanisms. Thus, traversing the landscape along pathway 2, in contrast to pathways 1 or 3 (Fig. 5), may not show the lowest fitness cost in a dynamic setting where, for example, the genotoxicity of the environment is fluctuating to a greater extent. Also, SOS network genes may acquire mutations that modify their inherent toxicity or regulation by LexA, which would in turn impact the fitness landscape. Furthermore, epistatic effects from other gene-environment interactions are likely to be significant given that the SOS response has additional layers of control in comparison to other stress response pathways. For example, the SOS response is impacted by the RpoS regulon (35–37), which mediates the generalized stress response and is activated by a variety of cellular stresses, including nutrient deprivation and entry into stationary-phase growth. Thus, the landscape derived here is limited to a snapshot under a specific set of experimental conditions, providing a framework for understanding the immediate fitness effects of changing *lexA*-NAR circuit parameters.

Finally, we included a comparison of the *lexA*^cons06^ mutant to the wild-type strain in order to understand the function of the double-operator configuration of the wild-type *lexA* promoter. We chose the cons06 promoter for this study not only because it contains only a single functional operator but also because *lexA*^cons06^ shares the same [LexA]_s_ and basal target gene expression levels as *lexA*^wt^, allowing a matched comparison of their circuit input-output features. It has been hypothesized that the double-operator structure of the wild-type *lexA* promoter may enable a cooperative binding mode that speeds up the shutoff kinetics of the *lexA*-NAR circuit (28). Modeling of cooperative binding of the TF in NAR predicts not only faster shutoff (7) but also a decrease in the input dynamic range (8). It has also been suggested that cooperative binding can promote “mutational robustness” and can potentially foster TF evolution by buffering against the fitness effects of deleterious TF gene mutations (20). In our analysis of the *lexA* promoter, however, we observed no evidence of interdimer cooperativity in the mode of repression. In agreement with past studies (22, 23), we found that operators I and II are equal in their abilities to repress *lexA* expression (see Fig. S1 in the supplemental material), but, comparing the levels of induction of the cons06 and wt promoters across a wide dose-range of DNA damage, we observed no detectable differences (Fig. S3). Furthermore, our analysis of circuit sensitivity, dynamic range, expression kinetics, and fitness in the fully integrated circuits of the *lexA*^cons06^ and *lexA*^wt^ strains also revealed no differences (Fig. 3 and 4). Although no formal biochemical analysis of interdimer cooperativity using purified components has been performed to our knowledge, our data show that the double-operator configuration has a negligible effect on transcriptional regulation compared to the single-operator configuration. Thus, the physiological role of the double-operator configuration, at the *lexA* promoter, remains unclear despite repeated speculation in the literature concerning cooperative binding (11, 20, 22, 23, 28). Of note, other SOS genes also contain multiple operators, such as *recN* (38) and the plasmid-borne colicin genes (39), but most SOS gene promoters contain a single operator. It is possible that one of the two operators of the *lexA* promoter also regulates input from an alternative TF, but no such factor has been discovered to date. Instead, we favor the idea that the double-operator configuration represents a fine-tuning mechanism for SOS regulation. For example, the E. coli *lexA* promoter also contains a Dcm methylase recognition site within operator I, and LexA binding is diminished when the DNA is methylated (28), but a regulatory role for this DNA modification has not been established. Interestingly, this Dcm site contains an “extended” sequence motif associated with hypomethylation during exponential-phase growth (40), suggesting a mechanism for differential regulation of the SOS response that is dependent on growth phase. Finally, studies of SOS activation kinetics in single cells have revealed more-complex behavior which is otherwise masked by signal averaging from aggregate cell populations (41). Thus, future investigations of these promoter features in single cells may be needed to fully delineate their specific impact on *lexA* regulation and *lexA*-NAR circuit behavior.

## MATERIALS AND METHODS

### Electromobility shift assay.

Recombinant LexA protein was overexpressed and purified as previously described (27). Protein concentrations were determined by Bradford assay. DNA oligonucleotides and 5′-IR700-modified oligonucleotides (see Table S2 in the supplemental material) were purchased from Integrated DNA Technologies, and DNA concentrations were determined by analysis of absorbance at 260 nm. Synthetic DNA operators were constructed by annealing the fluorophore-labeled oligonucleotide with a 1.2× molar excess of its complementary oligonucleotide using a thermocycler program with a slow ramp from 95°C to 25°C. Binding reactions were carried out at room temperature in a 20-μl volume. Reaction mixtures contained the indicated amount of LexA protein, 50 nM IR700-labeled operator DNA, 10 ng/μl sonicated salmon sperm DNA, 70 mM Tris-HCl (pH 7.6), 150 mM NaCl, 10 mM MgCl_2_, 1 mM dithiothreitol (DTT), and 5% glycerol. After incubation for 5 min, binding reaction mixtures were loaded onto a 6% native polyacrylamide gel and separated by electrophoresis in 0.5× Tris-borate-EDTA (TBE) buffer. Reaction products were visualized by scanning the gel with an Odyssey 9120 infrared imaging system (Li-Cor Biosciences).

10.1128/mSphere.00718-20.10 TABLE S2 DNA oligonucleotides used in this study. (A) Electromobility shift assays. Oligonucleotide sequences used to construct fluorescently labeled DNA probes are shown (/5IRD700/= 5′-IRdye 700). Bolded residue designations indicate CTG motifs, and underlined residues indicate T → C mutation of the CTG motif. (B) Site-directed mutagenesis. Two methods were used for site-directed mutagenesis. Where complementary primers were used (traditional method), the second primer is denoted as “reverse complement.” However, where nonoverlapping primers were used with a Q5 site-directed mutagenesis kit (New England Biolabs), both primer sequences are shown. Underlined residues indicate mutations. (C) Cloning and λ-red recombination. (D) Sequencing. Primer sequences used for Sanger sequencing are shown. Download Table S2, PDF file, 0.3 MB. Copyright © 2020 Kozuch et al. 2020 Kozuch et al. This content is distributed under the terms of the Creative Commons Attribution 4.0 International license.

### Bacterial strains and plasmids.

The DNA oligonucleotides used for construction of bacterial strains and plasmids in this study are listed in Table S2. The bacterial strains used in this study were all derivatives of the previously described E. coli K12 MG1655 *ΔsulA*::FRT (here, referred to as *lexA*^+^) and *ΔsulA*::FRT *ΔlexA::cat*-i-sceI (referred to here as “*ΔlexA*”) strains (19). To construct the *lexA* promoter mutant strains (referred to here as the *lexA*^wt^, *lexA*^2L2R^, *lexA*^2R1L^, *lexA*^2L2R1L1R^, *lexA*^cons11^, and *lexA*^cons06^ strains), this *ΔlexA* strain was transformed with pWRG99 in order to introduce specific mutations into the chromosomal *lexA* locus via λ-red-mediated recombination (42). To generate the double-stranded DNA (dsDNA) molecules for recombination, the *lexA* locus, along with approximately 1 kb of flanking sequence, was PCR amplified from wild-type MG1655 and cloned into pUC19 to make pBCK046. Then, the desired *lexA* promoter mutations were introduced into pBCK046 by site-directed mutagenesis. The resulting plasmids were used as templates for PCRs to prepare the linear dsDNA molecules for λ-red-mediated recombination. After electroporation of the dsDNA, the desired recombinants were selected by assessing loss of resistance to chloramphenicol. Mutations were confirmed by DNA sequencing. Strains were cured of pWRG99 by culture at 42°C and were evaluated for the loss of ampicillin resistance.

The GFP-reporter plasmids used in this study were derivatives of pUA66-P*_lexA_*-*gfp* from the E. coli promoter collection (GE Dharmacon) (26). Promoter mutations were introduced using site-directed mutagenesis, and the desired mutations were confirmed by DNA sequencing. The low-LexA-affinity (P_Lo_-*gfp*), high-LexA-affinity (P_Hi_-*gfp*), and no-LexA-affinity (P_No_-*gfp*) derivatives of pUA66-P*_recA_*-*gfp* used here have been previously described (27) and are referred to as the “TA,” “GG,” and “scram” operator derivatives there.

For *sulA*^+^ plasmid complementation, the *sulA* locus was cloned into the very-low-copy-number pUA66 vector to make pBCK059 (see Table S2). Vector DNA was prepared by digesting pUA66-P*_lexA_-gfp* with XhoI and XbaI, which removed both the *lexA* promoter and *gfp*. The desired product was confirmed by restriction analysis and DNA sequencing. Control transformations were carried out with pBCK005, which is also derived from pUA66 but lacks the *sulA* locus (see Table S2). pBCK005 was also used to control for nonspecific GFP background in promoter activity measurements (see below), as it lacks a promoter for *gfp* but retains the *gfp* gene.

### Quantitative Western blotting.

Protein samples were prepared from overnight cultures using BugBuster protein extraction reagent (Millipore). Samples were combined with Laemmli buffer, boiled, and separated by SDS-PAGE. Proteins were transferred to polyvinylidene difluoride (PVDF) membranes using an iBlot system (Thermo Fisher). LexA was probed using LexA (E-7) mouse monoclonal IgG_2b_ (1:200 dilution) as the primary antibody and horseradish peroxidase (HRP)-conjugated m-IgG_κ_ BP-HRP (1:2000) as the secondary antibody (Santa Cruz Biotechnology). LexA was detected by chemiluminescence using a ChemiDoc XRS+ imaging system (Bio-Rad). Relative LexA levels were quantified by interpolation of a standard curve that was constructed from a dilution series of a sample detected using the same exposure time. Background signal was determined using samples prepared from a *ΔlexA* strain. Relative loading levels were determined by quantifying total protein using Coomassie-stained SDS-PAGE gels. For experiments measuring LexA levels over time after UV-induced DNA damage, cells were grown in M9 minimal glucose media at 37°C and pulsed with UV light during mid-exponential phase. Then, aliquots of the culture were taken at the indicated times and processed immediately by pelleting the cells at high speed for 30 s, combining with Laemmli buffer, and boiling.

### GFP expression and promoter activity measurements.

GFP levels were measured in live cells growing in M9 minimal glucose media using an Infinite F Plex (Tecan) multifunction plate reader as previously described (27). Measurements in the absence of DNA damage were obtained during exponential growth. For each well, the fluorescence intensity value was normalized to cell density by dividing by the value representing the optical density at 595 nm (OD_595_), and then the logarithm (base 10) calculation was performed. To remove background GFP signal, values representing results from experiments performed using the pBCK005 control plasmid were subtracted.

Promoter activity kinetic measurements after different UV doses were performed and the results analyzed as previously described (27). Briefly, values obtained after taking the first derivative of the “GFP versus time” data were normalized by OD_595_ to yield promoter activity (PA) values with units of GFP/min/OD, which reflect the rate of GFP expression at any given time point. The largest PA value in the time trace is defined as the PA_peak_. PA_peak_ values were plotted as a function of the logarithm of the UV dose and then subjected to nonlinear regression analysis in Prism (GraphPad Software, Inc.) using a variable slope (four-parameter) dose-response model to determine the ED_50_ and R values. “PA versus time” traces were analyzed for differences in turn-on and shutoff kinetics as described in the text and figure legends. Averages of results from at least two independent experiments were used for analysis.

### UV sensitivity and rifampin mutation frequency.

Overnight cultures were diluted 1:100 into 75 ml of fresh M9 minimal glucose media and incubated at 37°C with shaking until they reached an OD_595_ of ∼0.4. Then, 10 ml of culture was irradiated with a UV dose of 40 J/m^2^ inside a 10-cm-diameter dish using a UVGL-58 (UVP) UV lamp set to 254 nm. The cultures were then placed in new tubes and allowed to recover by incubation at 37°C with shaking for 1 h. After this recovery period, aliquots were plated on LB agar for total population counts and LB-RIF agar (100 μg/ml rifampin (Rif) to score Rif-resistant (Rif^r^) mutants. Rif^r^ mutants were counted after 48 h of incubation. “No UV” control cells were treated in the same manner but were not irradiated.

### Growth rates.

Overnight cultures of each strain were diluted into fresh LB media by a factor of 10^6^, and growth was monitored by OD_595_ using an Infinite F Plex (Tecan) multifunction plate reader at 37°C with shaking. Experiments were performed in triplicate for each strain. Values and errors were determined for the maximum growth rate by fitting the growth curves to the Gompertz equation using nonlinear regression.

### Relative fitness.

To distinguish cells in coculture, we transformed the wild-type parental strain with plasmid pUA66-P_Lo_-*gfp* and each mutant strain with pBCK005 to create “GFP-bright” and “GFP-dark” strains, respectively. Overnight cultures of each strain were diluted into fresh LB media by a factor of 10^6^, mixed together in a 1:1 ratio, and incubated for 16 h at 37°C with shaking. The medium was supplemented with 60 μg/ml of kanamycin (Kan) for plasmid maintenance. In experiments measuring relative fitness levels in the presence of added genotoxic stress, 1 μg/ml MMC was added to the media. To determine the size of the initial populations (*N*_i_) and final populations (N_f_), aliquots of the coculture were plated on LB-Kan agar immediately after strain mixing and after the incubation period elapsed, respectively. GFP-bright and GFP-dark colonies were enumerated after overnight incubation using a blue light transilluminator. Relative fitness values (*W*) were calculated for each wild-type^bright^–mutant^dark^ strain combination and were then normalized to the wild-type^bright^–wild-type^dark^ result using the following equation:

$$W=\text{ln}\left(\frac{N_{\text{f}}^{\text{dark}}}{N_{\text{i}}^{\text{dark}}}\right)/\text{ln}\left(\frac{N_{\text{f}}^{\text{bright}}}{N_{\text{i}}^{\text{bright}}}\right)$$

### Relative fitness model of NAR circuit parameters.

For a generic NAR circuit, the repressor steady-state concentration, [*R*]_s_, can be expressed as a function of *k* and $\frac{\beta }{\alpha }$ (7) as follows:

(6) $${\left[\text{R}\right]}_{\text{s}}=\frac{\sqrt{{\text{k}}^{2}+4\text{k}(\frac{\beta }{\alpha })}-\text{k}}{2}$$

The 3D plots and color scales were constructed using MatLab (MathWorks) using a log_10_ transformation of equation 6. The color gradient of the plots generally correlates with the value of log_10_[*R*]_s_. Refinements of the color gradient along the log_10_[*R*]_s_ isoline were performed using Photoshop (Adobe Systems, Inc.).

### Statistical tests.

Statistical tests were performed with Prism (GraphPad Software, Inc.).
