# Supplementary figures and images for: The Parameter-Fitness Landscape of lexA Autoregulation in Escherichia coli
Source: mSphere. 2020 Aug 19;5(4):e00718-20. doi: 10.1128/mSphere.00718-20 (PMC7440846; doi:10.1128/mSphere.00718-20)

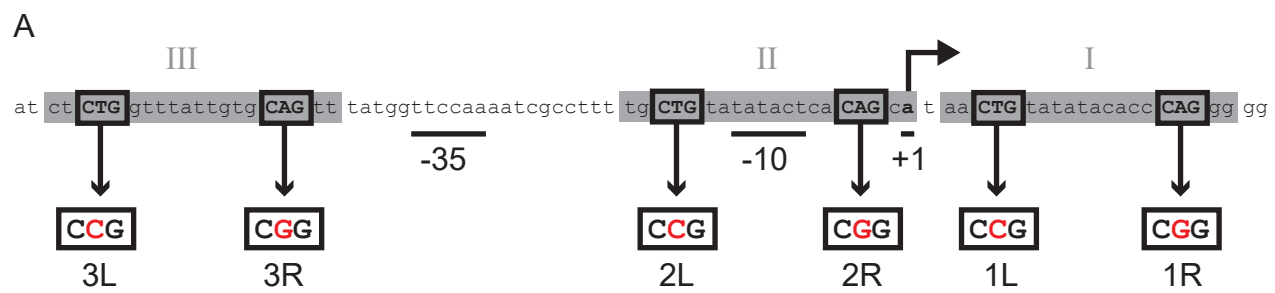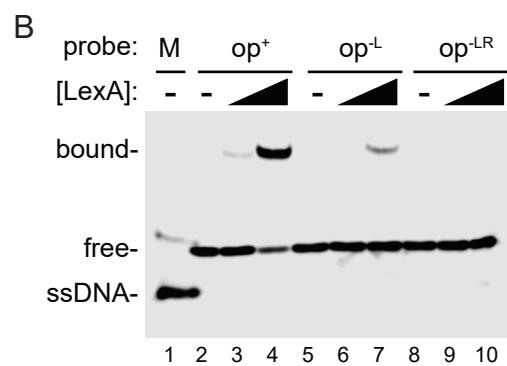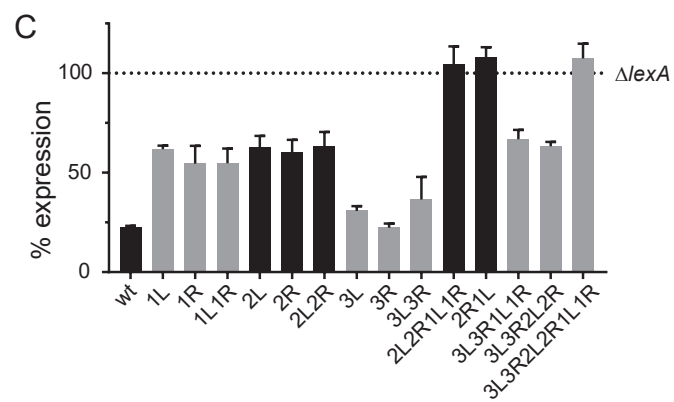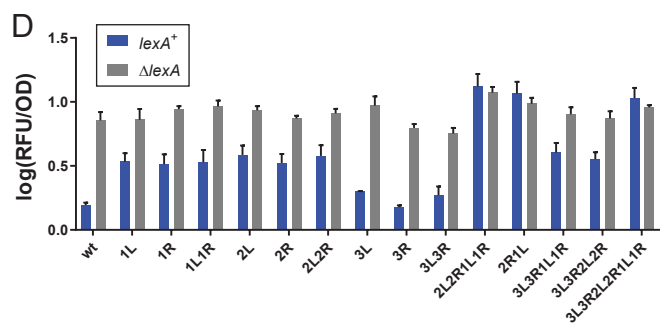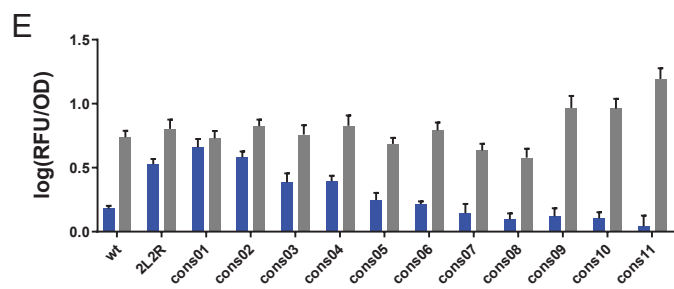

Supplement: FIG S1 [file mSphere.00718-20-sf001.pdf]

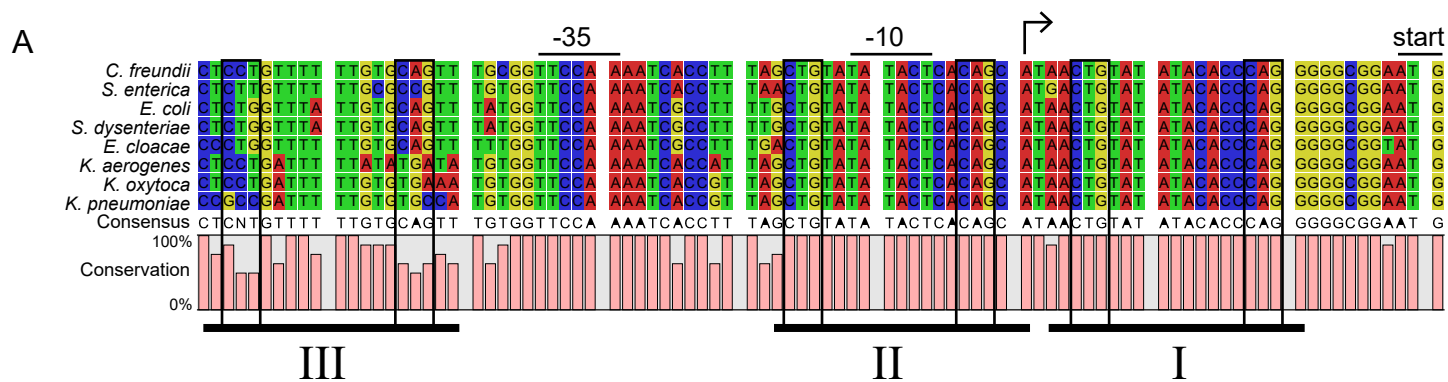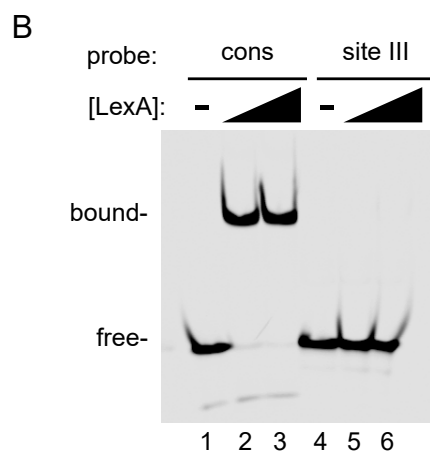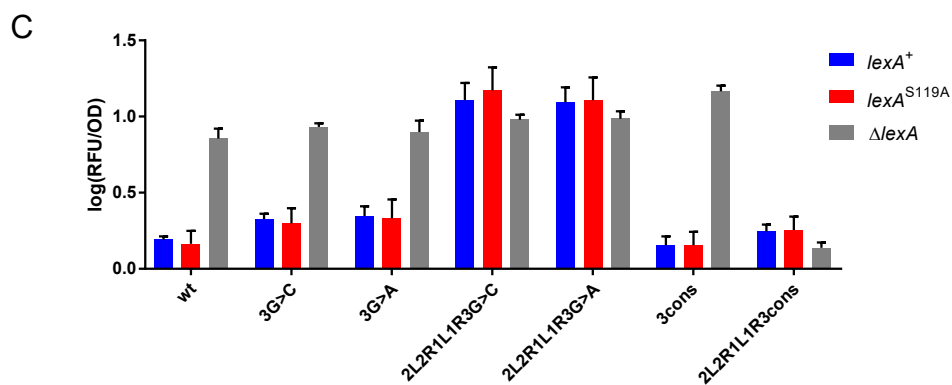

Supplement: FIG S2 [file mSphere.00718-20-sf002.pdf]

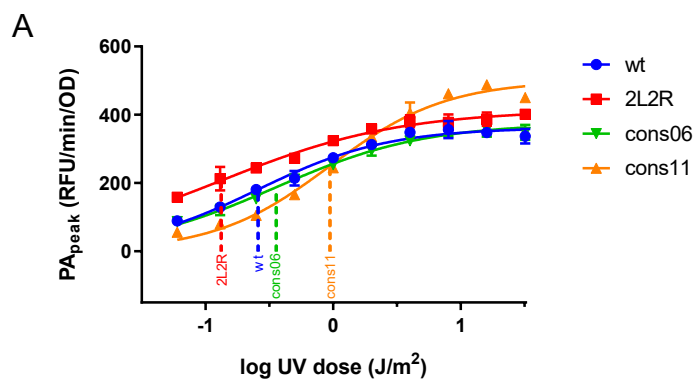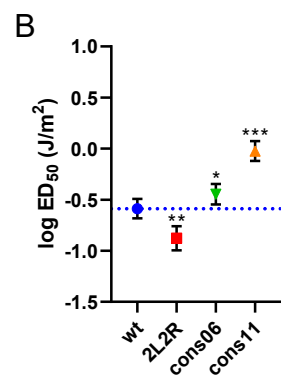

Supplement: FIG S3 [file mSphere.00718-20-sf003.pdf]

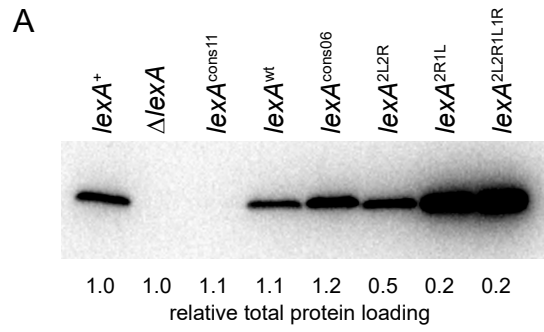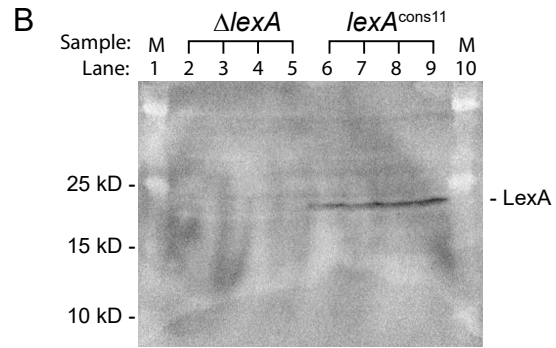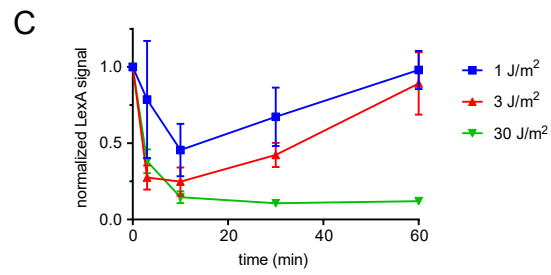

Supplement: FIG S4 [file mSphere.00718-20-sf004.pdf]

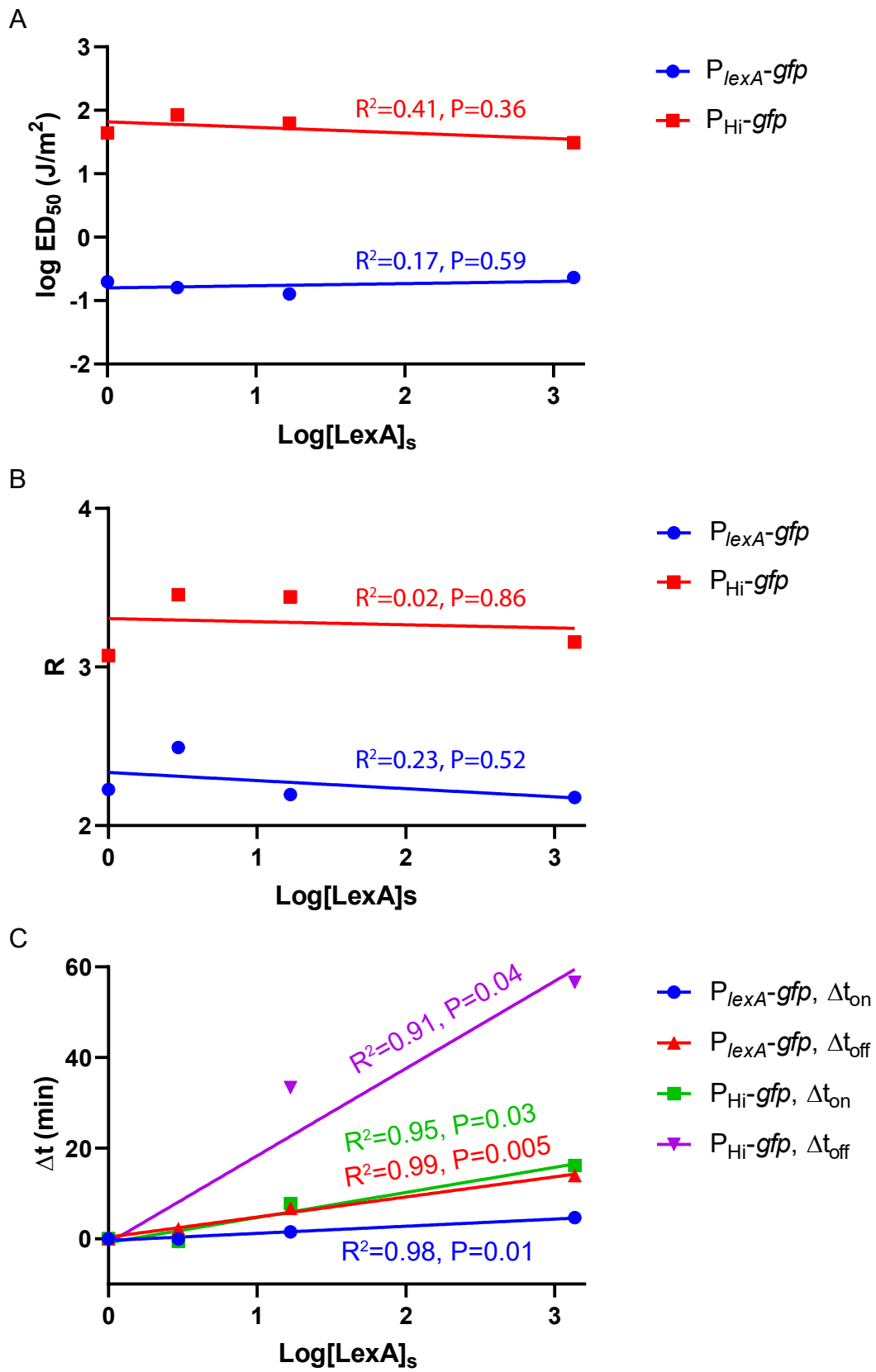

Supplement: FIG S5 [file mSphere.00718-20-sf005.pdf]

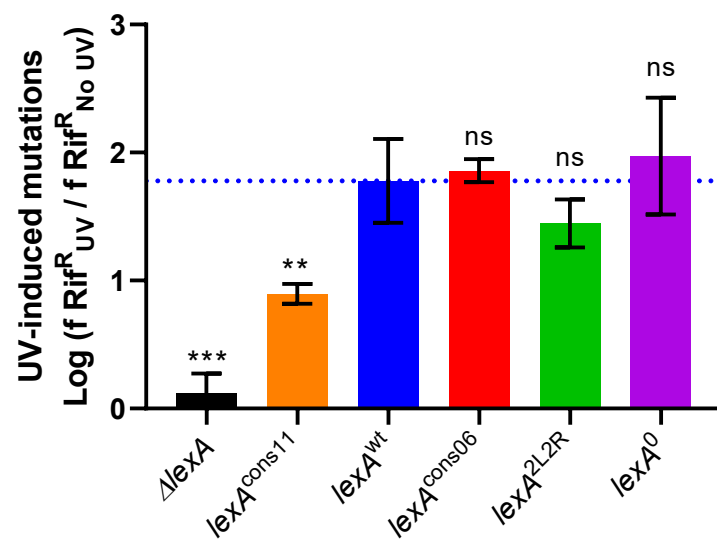

Supplement: FIG S6 [file mSphere.00718-20-sf006.pdf]

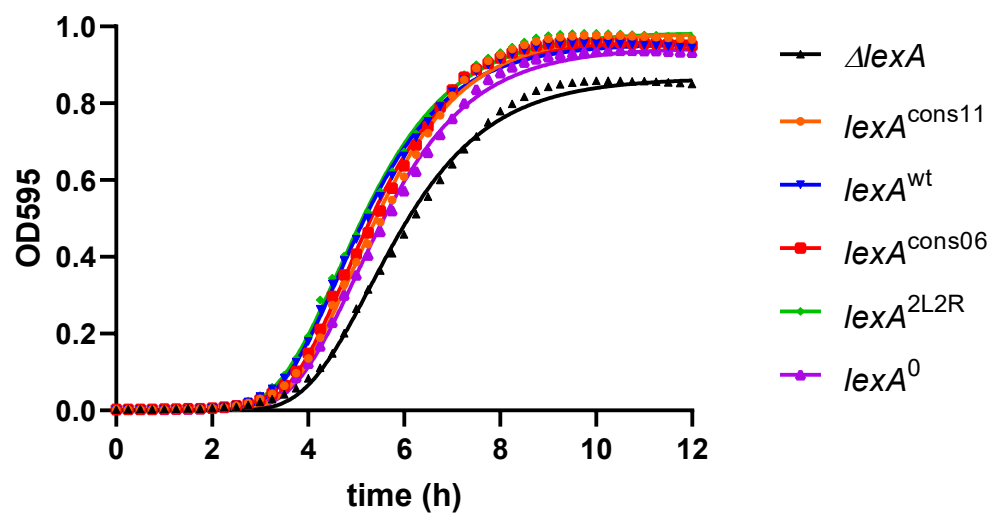

Supplement: FIG S7 [file mSphere.00718-20-sf007.pdf]

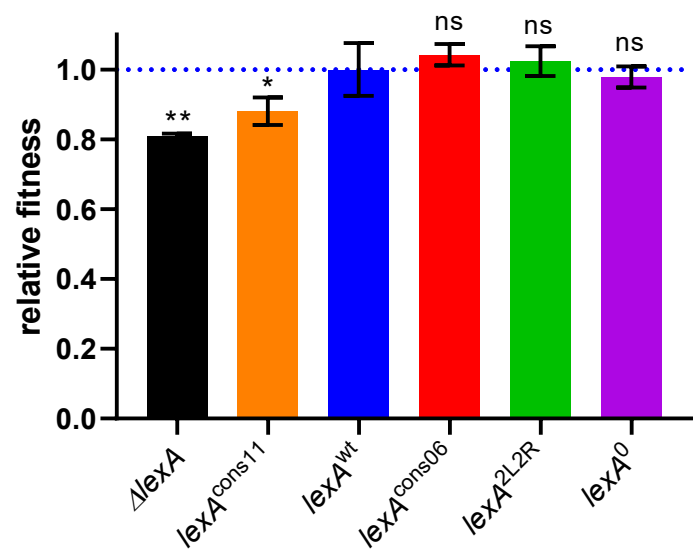

Supplement: FIG S8 [file mSphere.00718-20-sf008.pdf]
