# Supplementary material for: The Parameter-Fitness Landscape of lexA Autoregulation in Escherichia coli
Source: mSphere. 2020 Aug 19;5(4):e00718-20. doi: 10.1128/mSphere.00718-20 (PMC7440846; doi:10.1128/mSphere.00718-20)
Supplement: TABLE S1 [file mSphere.00718-20-st001.pdf]

Table S1

strain	pBCK005 (vector control)	pBCK059 (*sulA* $^{+}$)
lexA $^{+}$	816 ± 60	1001 ± 476
lexA ^{wt}	501 ± 42	994 ± 193
Δ *lexA*	360 ± 48	0 ± 0
lexA ^{cons11}	331 ± 14	0 ± 0
lexA ^{cons06}	423 ± 74	842 ± 91
lexA ^{2L2R}	478 ± 59	855 ± 312
