# Supplementary material for: The Parameter-Fitness Landscape of lexA Autoregulation in Escherichia coli
Source: mSphere. 2020 Aug 19;5(4):e00718-20. doi: 10.1128/mSphere.00718-20 (PMC7440846; doi:10.1128/mSphere.00718-20)
Supplement: TABLE S2 [file mSphere.00718-20-st002.pdf]

## Table S2. A. Electromobility shift assays.

| Probe | Description | Oligonucleotide sequences (5'→ 3') |
|---|---|---|
| op$^{+}$ (cons) | consensus operator sequence | /5IRD700/AATGCCTGCGGATA**CTG**TATATATATA**CAG**TATCAATTCTGGCT<br>AGCCAGAATTGATA**CTG**TATATATATA**CAG**TATCCGCAGGCATT |
| op$^{-L}$ | consensus operator sequence with one T>C mutation | /5IRD700/AATGCCTGCGGATA**CCG**TATATATATA**CAG**TATCAATTCTGGCT<br>AGCCAGAATTGATA**CTG**TATATATATA**CGG**TATCCGCAGGCATT |
| op$^{-LR}$ | consensus operator sequence with two T>C mutations | /5IRD700/AATGCCTGCGGATA**CCG**TATATATATA**CGG**TATCAATTCTGGCT<br>AGCCAGAATTGATA**CCG**TATATATATA**CGG**TATCCGCAGGCATT |
| site III | putative operator III sequence | /5IRD700/AATTCGATAAATCT**CTG**GTTTATTGTG**CAG**TTTATGGTTCCAAA<br>TTTGGAACCATAAA**CTG**CACAATAAAC**CAG**AGATTTATCGAATT |

## Table S2. B. Site-directed mutagenesis.

| Mutant | Plasmid Name | Template | Primer sequences (5'→ 3') |
|---|---|---|---|
| 1L | pBCK008 | pUA66-$P_{lexA}$-*gfp* | CTTTTGCTGTATATACTCACAGCATAACCGTATATACACCCAGG<br>reverse complement |
| 1R | pBCK002 | pUA66-$P_{lexA}$-*gfp* | TAACTGTATATACACCCGGGGGGCGGAATGAAAGC<br>reverse complement |
| 1L1R | pBCK007 | pUA66-$P_{lexA}$-*gfp* | GCATAACCGTATATACACCCGGGGGGCGGAATG<br>reverse complement |
| 2L | pBCK010 | pUA66-$P_{lexA}$-*gfp* | CCTTTTGCCGTATATACTCACAGCATAACTGTATATACACCCA<br>CGATTTTGGAACCATAAACTGCACAATAAACCAGAGATTTATC |
| 2R | pBCK003 | pUA66-$P_{lexA}$-*gfp* | CCTTTTGCTGTATATACTCACGGCATAACTGTATATACACCCA<br>reverse complement |
| 2L2R | pBCK013 | pBCK010 | CCTTTTGCCGTATATACTCACGGCATAACTGTATATACACCCA<br>CGATTTTGGAACCATAAACTGCACAATAAACCAGAGATTTATC |
| 3L | pBCK009 | pUA66-$P_{lexA}$-*gfp* | TAAATCTCCGGTTTATTGTGCAGTTTATGGTTCCAAAATCGCC<br>TCGAATTCTGGAAGGGTTATCCAAAATGCGACCATAACAGAATG |
| 3R | pBCK004 | pUA66-$P_{lexA}$-*gfp* | TAAATCTCTGGTTTATTGTGCGGTTTATGGTTCCAAAATCGCC<br>reverse complement |
| 3L3R | pBCK014 | pBCK009 | TAAATCTCCGGTTTATTGTGCGGTTTATGGTTCCAAAATCGCC<br>TCGAATTCTGGAAGGGTTATCCAAAATGCGACCATAACAGAATG |
| 2L2R1L1R | pBCK015 | pBCK013 | GCATAACCGTATATACACCCGGGGGGC<br>CGTGAGTATATACGGCAAAAGGCGATTTTGGAA |
| 2R1L | pBCK027 | pUA66-$P_{lexA}$-*gfp* | ATATACTCACGGCATAACCGTATATAC<br>ACAGCAAAAGGCGATTTTG |
| 3L3R1L1R | pBCK016 | pBCK014 | GCATAACCGTATATACACCCGGGGGGC<br>TGTGAGTATATACAGCAAAAGGCGATTTTGGAACC |
| 3L3R2L2R | pBCK018 | pBCK014 | ACTCACGGCATAACTGTATATACACCC<br>ATATACGGCAAAAGGCGATTTTGGAAC |
| 3L3R2L2R1L1R | pBCK017 | pBCK015 | TAAATCTCCGGTTTATTGTGCGGTTTATGGTTCCAAAATCGCC<br>TCGAATTCTGGAAGGGTTATCCAAAATGCGACCATAACAGAATG |
| 3G>C | pBCK023 | pUA66-$P_{lexA}$-*gfp* | GGTTTATTGTCCAGTTTATGGTTC<br>AGAGATTTATCGAATTCTGG |
| 3G>A | pBCK024 | pUA66-$P_{lexA}$-*gfp* | GGTTTATTGTACAGTTTATGGTTC<br>AGAGATTTATCGAATTCTGG |
| 2L2R1L1R3G>C | pBCK025 | pBCK015 | GGTTTATTGTCCAGTTTATGGTTC<br>AGAGATTTATCGAATTCTGG |
| 2L2R1L1R3G>A | pBCK026 | pBCK015 | GGTTTATTGTACAGTTTATGGTTC<br>AGAGATTTATCGAATTCTGG |

| | | | |
|---|---|---|---|
| 3cons | pBCK034 | pUA66-P$_{lexA}$-*gfp* | ATATACAGTATATGGTTCCAAAATCG<br>ATATACAGTAATTTATCGAATTCTGG |
| 2L2R1L1R3cons | pBCK035 | pBCK015 | ATATACAGTATATGGTTCCAAAATCG<br>ATATACAGTAATTTATCGAATTCTGG |
| cons01 | pBCK044 | pBCK013 | TGTATATACACCCAGGAGGCGG<br>GTTATGCCGTGAGTATATACGGCAAAAG |
| cons02 | pBCK043 | pBCK013 | TGTATATACACACAGGAGGCGG<br>GTTATGCCGTGAGTATATACGGCAAAAG |
| cons03 | pBCK039 | pBCK013 | TGTATATACACACAGGGGGCGG<br>GTTATGCCGTGAGTATATACGGCAAAAG |
| cons04 | pBCK040 | pBCK013 | TGTATATACACCCAGTGGGCGG<br>GTTATGCCGTGAGTATATACGGCAAAAG |
| cons05 | pBCK038 | pBCK013 | TGTATATATACCCAGGGGGCGG<br>GTTATGCCGTGAGTATATACGGCAAAAG |
| cons06 | pBCK041 | pBCK013 | TGTATATATACACAGGGGGCGG<br>GTTATGCCGTGAGTATATACGGCAAAAG |
| cons07 | pBCK042 | pBCK013 | TGTATATATATCCAGGGGGCGG<br>GTTATGCCGTGAGTATATACGGCAAAAG |
| cons08 | pBCK019 | pBCK013 | TGTATATACATCCAGGGGGCGG<br>GTTATGCCGTGAGTATATACGGCAAAAG |
| cons09 | pBCK020 | pBCK013 | TGTATATACATACAGGGGGCGG<br>GTTATGCCGTGAGTATATACGG |
| cons10 | pBCK021 | pBCK013 | ATATACAGGGGGCGGAATGAAAGCGTTA<br>ATATACAGTTATGCCGTGAGTATATACGGC |
| cons11 | pBCK022 | pBCK013 | ATATACAGTAGGCGGAATGAAAGCGTTA<br>ATATACAGTTATGCCGTGAGTATATACGGC |
| 2L2R | pBCK047 | pBCK046 | CCTTTTGCCGTATATACTCACGGCATAACTGTATATACACCCA<br>CGATTTTGGAACCATAAACTGCACAATAAACCAGAGATTTATC |
| cons06 | pBCK049 | pBCK047 | TGTATATATACACAGGGGGCGG<br>GTTATGCCGTGAGTATATACGGCAAAAG |
| cons11 | pBCK048 | pBCK047 | ATATACAGTAGGCGGAATGAAAGCGTTA<br>ATATACAGTTATGCCGTGAGTATATACGGC |
| 2L2R | pBCK060 | pBCK058 | CCTTTTGCCGTATATACTCACGGCATAACTGTATATACACCCA<br>CGATTTTGGAACCATAAACTGCACAATAAACCAGAGATTTATC |
| 2L2R1L1R | pBCK061 | pBCK060 | GCATAACCGTATATACACCCGGGGGGC<br>CGTGAGTATATACGGCAAAAGGCGATTTTGGAA |
| 2R1L | pBCK062 | pBCK058 | ATATACTCACGGCATAACCGTATATAC<br>ACAGCAAAAGGCGATTTTG |

## Table S2. C. Cloning and λ-red recombination.

| Plasmid Name | Description | Vector RE Digest | Insert PCR amplicon Primer sequences (5'→ 3'), Template DNA: MG1655 |
|---|---|---|---|
| pBCK005 | pUA66-*gfp* No promoter | pUA66-$P_{ftsK}$-*gfp* XhoI/BamHI | No insert: Overhangs filled with Klenow and blunt ends ligated |
| pBCK046 | pUC19-*lexA* | pUC19 XbaI/HindIII | TTTTTTTCTAGAGGTCAGGCAAGTCATGTG<br>TTTTTTAAGCTTCAGACCAATCAGCAATGTT |
| pBCK058 | pUA66-*lexA* | pUA66-$P_{lexA}$-*gfp* XbaI/XhoI | TTTTTTTCTAGAGGTCAGGCAAGTCATGTG<br>TTTTTTCTCGAGCAGACCAATCAGCAATGTT |
| pBCK059 | pUA66-*sulA* | pUA66-$P_{lexA}$-*gfp* XbaI/XhoI | TTTTTTTCTAGAGTCAGCGACAATTTACAG<br>TTTTTTCTCGAGTCGGTATTCAATTGTGCC |
| For PCR amplification of *lexA* locus from pBCK046 and pBCK058 derivatives (for λ-red recombination) | | | GGTCAGGCAAGTCATGTG<br>CAGACCAATCAGCAATGTT |

## Table S2. D. Sequencing.

| Description | Primer sequences (5'→ 3') |
|---|---|
| For sequencing *lexA* promoter in pUA66-$P_{lexA}$-*gfp* derivatives | CATCACCATCTAATTCAACAAGAATTG |
| For sequencing *lexA* promoter in pBCK046 and pBCK058 derivatives | GCTGGTGATGATTGTGGA |
| For sequencing *lexA* in recombinant strains | TTACAGCCAGTCGCCGTTG |
| For sequencing *lexA* insert in pBCK046 | AGCGAGTCAGTGAGCGAG<br>GTTTTCCCAGTCACGAC (M13-40FOR)<br>CGGATAACAATTTCACACAG (M13-48REV) |
| For sequencing inserts in pBCK058 and pBCK059 | TAGCAACACCAGAACAGC |
